# COVID‐19 and pulmonary fibrosis: A potential role for lung epithelial cells and fibroblasts

**DOI:** 10.1111/imr.12977

**Published:** 2021-05-24

**Authors:** Alison E. John, Chitra Joseph, Gisli Jenkins, Amanda L. Tatler

**Affiliations:** ^1^ Nottingham NIHR Respiratory Biomedical Research Centre University of Nottingham Nottingham UK; ^2^ National Heart and Lung Institute Imperial College London UK

**Keywords:** COVID‐19, epithelial cells, fibroblasts, lung fibrosis, lung remodeling, SARS‐CoV‐2

## Abstract

The COVID‐19 pandemic rapidly spread around the world following the first reports in Wuhan City, China in late 2019. The disease, caused by the novel SARS‐CoV‐2 virus, is primarily a respiratory condition that can affect numerous other bodily systems including the cardiovascular and gastrointestinal systems. The disease ranges in severity from asymptomatic through to severe acute respiratory distress requiring intensive care treatment and mechanical ventilation, which can lead to respiratory failure and death. It has rapidly become evident that COVID‐19 patients can develop features of interstitial pulmonary fibrosis, which in many cases persist for as long as we have thus far been able to follow the patients. Many questions remain about how such fibrotic changes occur within the lung of COVID‐19 patients, whether the changes will persist long term or are capable of resolving, and whether post‐COVID‐19 pulmonary fibrosis has the potential to become progressive, as in other fibrotic lung diseases. This review brings together our existing knowledge on both COVID‐19 and pulmonary fibrosis, with a particular focus on lung epithelial cells and fibroblasts, in order to discuss common pathways and processes that may be implicated as we try to answer these important questions in the months and years to come.

## 1 SARS‐COV‐2 AND COVID‐19

Early in December 2019 reports of a pneumonia‐like illness of unknown etiology began to emerge in Wuhan City, Hubei province, China. The cause was rapidly identified as a novel coronavirus, initially named 2019‐nCoV, belonging to the genus betacoronavirus, which includes the SARS‐CoV and MERS‐CoV viruses that cause severe acute respiratory syndrome (SARS) and Middle East respiratory syndrome (MERS) respectively.^1^ Phylogenetic analysis of the virus demonstrated that it shared 79% sequence identity with SARS‐CoV ^2^ and therefore while it was closely related it was indeed a distinct virus, which was named by the International Committee on Taxonomy of Virus as SARS‐CoV‐2. The World Health Organisation (WHO) subsequently named the disease caused by SARS‐CoV‐2 as COVID‐19 on February 11, 2020. The virus spread rapidly and by March 11, 2020 the World Health Organisation declared a global pandemic. At the time of writing ( April 26, 2021) there have been greater than 147m cases of COVID‐19 and more than 3.1m deaths worldwide.

The severity of COVID19 ranges from asymptomatic infection, through mild flu‐like symptoms, to severe COVID19 that can rapidly progress to respiratory distress requiring intensive care treatment and mechanical ventilation and can ultimately result in respiratory failure and death. Recent studies have shown that the infection fatality rate (IFR) from COVID‐19 varies substantially across geographical locations, which may reflect the variation in population age.^3^, ^4^ Increased age is a major contributing factor to mortality from COVID‐19.^3^, ^5^ Furthermore, increased age is associated with higher risk of hospitalization following COVID19 infection.^6^ In addition to increased age, various other factors are now well documented to increase risk of death from COVID‐19 including gender (males have higher mortality), ethnicity, obesity, and pre‐existing medical conditions including diabetes, chronic respiratory, cardiac and liver diseases, reduced kidney function, hematological malignancies, and neurological diseases.^7^

## 2 PATHOGENESIS OF COVID‐19

Although COVID‐19 affects multiple organ systems including the cardiovascular and gastrointestinal systems, the respiratory system is the primary site of SARS‐CoV‐2 pathology.^8^ SARS‐CoV‐2 is transmitted through respiratory droplets and aerosols and is likely initially received by the epithelium of the nasopharynx in the first instance, where active viral replication occurs early in the course of the disease.^9^ As the disease progresses, infection of lung epithelium occurs. The transmission of virus from the nasopharynx to the lung epithelium is supported by data showing that viral load peaks much earlier in throat swabs than in sputum samples; viral load likely peaks around the time of symptom onset in throat swabs but after onset of symptoms in sputum.^9^ Crucially, higher sputum viral loads and prolonged viral shedding in the lungs are associated with COVID‐19 severity,^10^, ^11^ suggesting that more efficient transmission of the virus from the upper respiratory tract (URT) to the lower respiratory tract (LRT) may contribute to the severity of symptoms.

In the immediate search for a cellular receptor that mediates SARS‐CoV‐2 viral entry, much work focused upon angiotensin converting enzyme 2 (ACE2), which has been shown to mediate SARS‐CoV viral entry ^12^, ^13^, ^14^, ^15^ and is highly expressed in the nasal epithelium.^14^ Single cell RNA sequencing (scRNAseq) has shown that ACE2 is differentially expressed within the respiratory tract with high levels in the nasal epithelium, the initial site of infection, lower expression in the tracheal and bronchial epithelium, and only 1.2% of alveolar type 2 epithelial (AT2) cells expressing ACE2 transcripts.^16^ The receptor binding domain (RBD) of SARS‐CoV‐2 is found within the S1 subunit of the spike protein.^17^ While sequence alignment studies have shown 76% similarity between SARS‐CoV and SARS‐CoV‐2, the S1 protein is not well conserved with only 64% identity between the two viruses, which may explain the significant difference in transmissibility between the two viruses ^18^ through as yet undefined co‐receptors. Following binding of the RBD to ACE2, the S1 protein is primed by proteolytic cleavage mediated by transmembrane protease serine 2 (TMPRSS2), ^14^ which facilitates fusion of the viral and cell membranes to allow viral entry.

In addition to ACE2, numerous other putative receptors for viral entry have been suggested including Cathepsin L1, CD147 and GRP78.^19^ Crucially, recent studies have highlighted a number of novel receptors that can bind SARS‐CoV‐2 including integrins αvβ3 and αvβ6,^20^ low‐density lipoprotein receptor class A domain containing 3 (LDLRAD3) and C‐type lectin domain family 4 member G (CLEC4G).^21^ The pathological consequences of SARS‐CoV‐2 binding to such receptors in vivo are yet to be confirmed, however, viral entry to proteins other than ACE2 may help to explain (a) the difference in transmissibility and disease severity between SARS‐CoV and SARS‐CoV‐2, and (b) the multi‐organ nature of COVID‐19 pathology.

Within the lungs COVID‐19 infection can be broadly divided into three main phases: an early infection phase involving viral replication and relatively mild symptoms; a second pulmonary phase characterized by stimulation of adaptive immunity and predominance of respiratory dysfunction as a result of lung injury and hypoxemia, and finally in patients who develop the most severe disease, a third systemic hyperinflammation phase.^22^ In these patients, direct viral injury, uncontrolled cytokine release, and microvascular inflammation can combine to cause multi‐organ failure, acute respiratory distress syndrome (ARDS), hemorrhage/coagulopathy, and secondary bacterial infections.^23^, ^24^, ^25^

Histologically, patients with COVID‐19 present with three main patterns: (i) epithelial with reactive epithelial changes and diffuse alveolar damage (DAD); (ii) vascular with microvascular damage, (micro)thrombi, and acute fibrinous and organizing pneumonia and (iii) fibrotic with evidence of interstitial fibrosis. The epithelial and vascular patterns may present alone, simultaneously or consecutively in all stages of symptomatic COVID‐19 with epithelial damage and vasculopathy characteristic of the early infection while fibrotic changes occur later, generally 3 weeks after symptoms.^26^ Two distinct patterns of fatal COVID19 disease with differing clinical courses have been suggested: one characterized by high viral load and high cytokine expression in the lungs but limited morphological changes and a second characterized by low viral load and cytokine expression but elevated numbers of immune cells (including CD8+ T cells and macrophages), which correlate with the presence of DAD.^27^

As the global COVID‐19 pandemic has progressed, a large number of patients have reported a range of symptoms persisting beyond the period of acute infection and illness. A range of studies have identified persistent fatigue in 60% and breathlessness in 40% of people up to 3 months following discharge from hospital. ^28^ Early lung function and radiology assessments are consistent with impaired pulmonary perfusion, alveolar scarring consistent with respiratory problems including fibrotic lung disease, bronchiectasis, and pulmonary vascular disease.^29^ Much of the evidence for these possible sequelae are derived from the early data in COVID‐19 patients along with extrapolation of data from previous SARS and MERS outbreaks and patients with ARDS.^30^, ^31^, ^32^, ^33^ However, a recent systematic review has shown that approximately 20% of COVID‐19 patients had evidence of fibrotic sequelae that persisted at 1‐year follow‐up,^34^ suggesting that fibrotic changes did not resolve over this time period. Furthermore, approximately 45% of COVID‐19 patients had impaired diffusing capacity for carbon monoxide (DLC) at follow‐up.^34^ The presence of ongoing symptoms in COVID‐19 patients has been termed long COVID or post‐COVID‐19 syndrome and although prospective studies are required in order to fully evaluate the population morbidity and consequences of these clinical manifestations, given the high case volume worldwide they pose a growing and significant health concern.

## 3 CURRENT UNDERSTANDING OF PULMONARY FIBROSIS

Pulmonary fibrosis is characterized by excessive extracellular matrix (ECM) deposition within the lung interstitium and destruction of the normal parenchymal structure leading to progressive loss of pulmonary function. It is a key feature of a variety of interstitial lung diseases (ILDs) of which idiopathic pulmonary fibrosis (IPF) is the most severe and has the worst mortality. IPF is a progressive, incurable disease with survival rates worse than most cancers.^35^ Treatment options are limited to two clinically approved drugs, Nintedanib (Ofev) and pirfenidone (Esbriet), both of which slow progression but do not halt or reverse the fibrosis.^36^, ^37^, ^38^, ^39^ Ultimately, the vast majority of IPF patients succumb to respiratory failure and death.^40^

In recent years, great advances have been made in our understanding of the underlying pathogenesis of pulmonary fibrosis. A combination of genetic, environmental and aging factors is involved in the initiation of the fibrotic processes, which likely begins many years before clinical manifestations become apparent.^41^ Genome‐wide studies have highlighted numerous genes associated with the development of IPF including *MUC5B, TERT, FAM13A, DSP and AKAP13* among others.^42^, ^43^, ^44^ Recent evidence shows that over 17% of non‐familial IPF cases in the over 65s can be attributed to a known genetic susceptibility variant ^45^, ^46^ Furthermore, genetic variants in genes associated with telomere length or surfactant function have been found in cases of familial pulmonary fibrosis.^47^, ^48^ Environmental factors including smoking, dust inhalation and asbestos exposure are also associated with increased risk of IPF,^46^ which on a backdrop of genetic susceptibility, contribute to IPF development. Infection, particularly from viruses, has also been postulated to contribute to the development of pulmonary fibrosis and with acute exacerbations of the disease.^49^

The pathogenesis of pulmonary fibrosis involves repeated microinjury to the alveolar epithelium that leads to an aberrant and ineffective repair response and epithelial dysfunction, which results in the transdifferentiation, activation and expansion of fibroblasts/myofibroblasts (reviewed in ^50^ ). Physiological repair of injured alveolar epithelial cells is facilitated by signals from underlying (myo)fibroblasts.^51^ Once the wound is effectively repaired myofibroblasts numbers dramatically reduce through a combination of apoptosis, senescence and reverse differentiation.^52^ The failure to terminate the wound‐healing response once the injury has been effectively repaired is characteristic of fibrosis and leads to the excessive production and deposition of ECM proteins by myofibroblasts.^53^ In addition, changes in the balance between matrix metalloproteinases (MMPs) and tissue inhibitors of MMPS (TIMPs) which ordinarily tightly regulate collagen turnover, enhanced extracellular matrix (ECM) crosslinking, and changes in immune cell numbers and phenotype can all contribute to alterations in the interstitial ECM structure and composition in pulmonary fibrosis.^54^, ^55^, ^56^, ^57^, ^58^ Parenchymal fibrosis leads to stiffening of the lung tissue,^59^ which dramatically impairs lung function by restricting total lung capacity and forced vital capacity.^60^ Crucially, stiffening of the lung tissue perpetuates the fibrotic response.^59^, ^61^, ^62^, ^63^

While changes to the structure and mechanical properties of the matrix drive lung fibrosis and contribute to its progressive nature, the two key cell types involved in the initiation of fibrogenesis are the alveolar epithelial cells and fibroblasts. Here we will discuss the relative contributions of each cell type to the pathogenesis in more detail.

## 4 EPITHELIAL CELLS: THE INITIATOR

The alveolar epithelium is comprised of type I and type II pneumocytes otherwise known as alveolar epithelial cells (ATI and ATII, respectively). ATI cells are thin, squamous cells that form the alveolar structures and are the site of gas exchange. ATII cells are cuboidal and more numerous than ATI cells, however, are significantly smaller in size. They contain large numbers of lamellar bodies and are responsible for the production and secretion of surfactant, which is critical to normal lung function. Furthermore, ATII cells, unlike ATI cells, are capable of proliferating and differentiating in to ATI cells, and act as progenitor cells during repair of damage to the alveolar epithelium.^64^

The alveolar epithelium is the site of initial injury early in the pathogenesis of IPF and it is thought that the loss of ATII cells, which is evident in IPF,^65^ is critical as loss of ATII cells can initiate fibrogenesis.^66^, ^67^ The injury initiates dramatic changes within the alveolar epithelium; the regenerative capacity of ATII cells, which during normal repair proliferate and differentiate in to ATI cells to restore alveolar integrity,^68^ is lost and cells acquire markers of airway epithelial cells in a process termed bronchiolarization,^69^ and the damaged alveolar epithelium undergoes apoptosis.^70^, ^71^ Furthermore, there is emerging evidence that ATII cell senescence contributes to IPF pathogenesis.^67^

In response to the injury, the alveolar epithelium releases a diverse array of soluble mediators, inflammatory cytokines and pro‐remodeling factors that have been implicated in the pathogenesis of IPF. Most notably epithelial cells can activate the potently pro‐fibrotic cytokine, transforming growth factor‐β, which is crucial to the pathogenesis of pulmonary fibrosis,^53^ through cell surface integrins, including αvβ6 integrins.^72^, ^73^ Once activated, TGFβ acts upon the underlying mesenchyme to stimulate fibrogenesis (for more details see subsequent section).^53^ Additionally, TGFβ upregulates expression of αvβ6 integrins as part of a positive feedback loop that promotes progressive fibrogenesis.^74^, ^75^ Furthermore, alterations in pathways that impact epithelial cell activation of TGFβ can lead to the development of spontaneous age‐related lung fibrosis in vivo.^76^

In addition to TGFβ, the damaged alveolar epithelium releases numerous other soluble factors known to be involved in pulmonary fibrosis. Increased platelet derived growth factor (PDGF) release from the epithelium has been described ^77^ and inhibition of PDGF signaling with receptor tyrosine kinase inhibitors or blocking antibodies reduces experimental lung fibrosis.^78^, ^79^ Crucially, one of the two clinically approved drugs for IPF, Nintedanib, acts in part through inhibition of PDGF signaling. Connective tissue growth factor (CTGF) is an additional growth factor that is released by ATII cells in IPF,^80^ the blockade of which can reduce radiation‐induced lung fibrosis.^81^ Furthermore, overexpression of CTGF is sufficient to induce a transient fibrotic response in the rat lung.^82^ ATII cells also secrete a milieu of inflammatory cytokines. Interluekin‐6 (IL6), a key pro‐inflammatory cytokine, is released from ATII cells and its expression is increased in the hyperplastic alveolar epithelium in pulmonary fibrosis.^83^ Furthermore, blockade of IL6 signalling in an in vivo mouse model abrogates pulmonary fibrosis.^84^

## 5 FIBROBLASTS: THE EFFECTOR

Lung fibroblasts play a pivotal role in the development and progression of lung fibrogenesis. The reside in relatively small numbers within the normal lung interstitium, however, in response to injury become activated to mediate wound repair. During normal wound‐healing responses, fibroblasts proliferate and transdifferentiate in to contractile, matrix producing myofibroblasts in order to construct new ECM to support new cells and contract the wound, after which the cells apoptose to resolve the wound‐healing response.^85^ However, in pathological fibrosis the repair response does not resolve, myofibroblasts persist and continue to deposit matrix proteins within the lung interstitium.^86^

In the context of lung fibrosis, fibroblasts are primarily activated through their close interaction with the injured alveolar epithelium. The vast array of secreted proteins from the injured epithelium has profound effects upon the underlying mesenchymal cell population. TGFβ, activated by the alveolar epithelium in response to injury, causes fibroblast proliferation,^87^, ^88^ transdifferentiation to a contractile myofibroblast phenotype,^89^, ^90^, ^91^, ^92^ and induces the production and deposition of ECM proteins.^87^, ^93^ Overexpression of TGFβ in vivo drives fibroblast proliferation, myofibroblast transdifferentiation and progressive lung fibrosis,^94^ highlighting the crucial role of TGFβ‐mediated effects on fibroblasts in IPF. Importantly, contraction of myofibroblasts can result in TGFβ activation further perpetuating pro‐fibrotic signals.^95^

Growth factors released by the injured alveolar epithelium impact fibroblast pro‐fibrotic responses and contribute to the development and progression of pulmonary fibrosis. PDGF is a potent mitogen for lung fibroblasts ^79^, ^96^, ^97^ and blockade of PDGF signaling, specifically that mediated through PDGF receptor‐beta, can reduce lung fibrosis in an experimental bleomycin mouse model.^79^ Similarly, CTGF stimulates fibroblast proliferation,^81^, ^98^ migration,^99^ and also increases collagen production.^100^

Pro‐fibrotic responses of fibroblasts are also profoundly influenced by inflammatory cytokines. Interleukin‐1 (IL1) overexpression in vivo initiates a dramatic pro‐inflammatory state indicative of acute lung injury (ALI) that results in severe, progressive pulmonary fibrosis.^101^ IL6 acts as a mitogen for fibroblasts isolated from fibrotic lung tissue ^102^ and Wnt1‐inducible signaling protein 1 (WISP1)‐induced fibroblast proliferation is mediated by IL6.^103^ Moreover, IL6 can reduce apoptosis in fibrotic lung fibroblasts.^104^ Interleukin‐11 (IL11) contributes to fibroblasts transdifferentiation in to myofibroblasts and stimulates collagen production via an extracellular signal‐regulated kinase (ERK)‐dependent pathway.^105^ Interleukin‐25 (IL25) enhances fibroblasts proliferation and production of collagens, and augments the release of CTGF from alveolar epithelial cells.^106^ Additionally, the Th17 cytokine interleukin‐17 (IL17) also increases fibroblast proliferation and collagen production.^107^ While the relative role of inflammation in the development and progression of pulmonary fibrosis is somewhat controversial, it is clear that inflammatory signaling pathways are capable of driving a pro‐fibrotic phenotype in lung fibroblasts.

## 6 PULMONARY VIRAL INFECTION AND FIBROSIS

The development of pulmonary fibrosis is often reported as an important sequelae to severe or persistent lung damage including in patients with respiratory infections,^49^ connective tissue disorders ^108^ and chronic granulomatous disease.^109^ Fibrosis is also a known sequelae of Acute Respiratory Distress Syndrome ^110^ and although many ARDS patients survive the acute phase of the illness, a substantial proportion of patients who have a longer disease duration (>3 weeks) will die as a result of progressive pulmonary fibrosis.^111^

Although a direct relationship between respiratory viral infection and development of progressive fibrosis has not been fully established, evidence from the previous global SARS outbreaks with SARS‐CoV and Middle East Respiratory Syndrome (MERS) shows a clear link between coronavirus infection, persistent impairment of lung function and abnormal radiological findings consistent with pulmonary fibrosis.^31^, ^32^, ^33^ Other respiratory viruses including Influenza H1N1 and H5N1 are also proposed to promote the development of pulmonary fibrosis ^112^, ^113^, ^114^, ^115^ while Hepatitis C, human cytomegalovirus and Epstein–Barr virus may act as viral cofactors in the development of IPF.^116^

The most extensive evidence of respiratory viral infection leading to fibrotic changes in the lung, is from a number of prospective studies of patients with Severe Acute Respiratory Syndrome (SARS) or Middle East Respiratory Syndrome (MERS). These reports show that 30%‐60% of patients exhibit impairment of lung function following infection in addition to evidence of parenchymal abnormalities.^31^, ^32^, ^33^, ^117^, ^118^ Follow‐up studies document that these lung abnormalities persist for many months postinfection with a gradual improvement in pulmonary function being seen over months to years in SARS patients.^118^, ^119^

In the longest reported follow‐up study, serial CT scans in 71 SARS infected patients between 2003 and 2018 reveal parenchymal abnormalities including ground glass opacities and cord‐like consolidation in 27 patients (38%).^120^ Assessment of the percentage of lung area containing lesions over the 15‐year period in these patients shows a significant reduction within the first 12 months after infection (from 9.40% in 2003 to 3.20% in 2004). However, the fibrotic changes persist and remain stable over subsequent years with lesions detected in 4.6% of the lung in 2018 with one patient exhibiting obstructive lung disease.^120^

## 7 COVID‐19 INFECTION, ARDS AND FIBROSIS

Due to the rapid increase in fatalities following infection with SARS‐CoV‐2, evidence of an association between viral infection and the development of pulmonary fibrosis in COVID‐19 patients appeared early in the pandemic with the degree of fibrotic change ranging from fibrosis in organizing pneumonia to widespread fibrotic disease following severe acute lung injury.^121^ The diagnosis of COVID‐19‐associated ARDS in patients with severe disease,^24^, ^122^ evidence of extensive pulmonary interstitial fibrosis in explanted lungs from COVID‐19 patients receiving lung transplants for end‐stage ARDS,^123^ and the presence of DAD, thickening of alveolar septa, proliferation of fibroblasts and evidence of fibrosis in other postmortem analyses confirmed this link.^124^, ^125^, ^126^, ^127^, ^128^

In addition to the histological findings in postmortem COVID‐19 lung tissue, radiological evidence of fibrosis is seen in chest CT scans of both symptomatic and asymptomatic patients following SARS‐CoV‐2 infection.^128^, ^129^, ^130^, ^131^ The key radiological features of COVID‐19 infection are bilateral distribution of ground glass opacities (GGO) with or without consolidation in posterior and peripheral lungs.^132^ In early studies, the extent of lung abnormalities detected by CT scan showed a marked increase from the subclinical period (0‐7 days) through the first and second weeks after symptom onset before decreasing gradually into week three.^128^, ^130^, ^131^, ^133^ These findings mirror the chronology of fibrosis detected in patients with ARDS.^134^

As previously seen with SARS and MERS, many COVID‐19 patients followed by serial CT imaging during hospitalization show significant radiological improvement at the time of discharge. However, for a proportion of patients, radiologic deterioration is linked to a poor prognosis.^128^, ^135^ Although it is still too early to determine whether the COVID19‐associated fibrotic changes in the lung are irreversible, recent evidence confirms persistent functional and radiological respiratory abnormalities at 4 months ^136^ and 6 months ^137^ after severe COVID‐19 illness. In addition, a recent systematic review and meta‐analysis investigating the prevalence of radiological and functional consequences posthospitalization for viral pneumonitis reports that although on follow‐up, the inflammatory consequences and features of fibrosis in COVID‐19 patients are reduced from baseline, fibrotic sequelae are still observed in a similar proportion of people across different follow‐up times. These data suggest that in SARSCoV2 infection as with SARS and MERS, the pulmonary fibrosis associated with viral pneumonitis does not resolve substantially in the first year following infection.^34^

Although it is too early to determine whether COVID‐19 patients exhibiting significant lung abnormalities postinfection will ultimately develop stable, low levels of pulmonary fibrosis with relatively normal lung function as with SARS or MERS, or go on to develop progressive pulmonary fibrosis, it is likely that even long‐term residual pulmonary fibrosis will result in significant morbidity particularly in older patients with other co‐morbidities.^138^ Shojaee et al (2021) recently reported that in two retrospective observational cohort studies using longitudinal hospitalization records, viral pneumonia is associated with an increased risk of developing postinflammatory pulmonary fibrosis (PIPF) and that patients with a prior viral pneumonia diagnosis developed PIPF earlier and at a younger age.^139^ Given the scale of the global COVID‐19 pandemic, the number of people requiring invasive ventilation, and the degree of lung injury in these patients, it is likely that the incidence of postviral fibrosis will increase substantially in the coming years. Understanding the mechanisms underlying COVID‐19 mediated lung fibrosis may therefore be key to developing targeted strategies for treating patients with Long COVID or post‐COVID‐19 syndrome.

## 8 MECHANISM OF COVID‐19 MEDIATED PULMONARY FIBROSIS: THE STORY SO FAR

### 8.1 Role of epithelial cells

The airway epithelial layer is a pseudostratified mucosal barrier comprising several cell types, which acts as a barrier to many pathogens such as SARS‐CoV‐2, MERS‐CoV (Middle East respiratory syndrome‐related coronavirus), and SARS‐CoV.^140^ Although Angiotensin‐converting enzyme 2 (ACE2) is reported to be the primary receptor for SARS‐CoV‐2, its contribution to SARS‐CoV‐2 infectivity is not well understood. Despite reports of very low levels of ACE2 expressing cells in the alveolar parenchyma SARS‐CoV‐2 infection leads to substantial alveolar damage.^141^ In response to injury, including following viral infection, AT2 cells which are generally more injury‐resistant migrate to the damaged area of lung, differentiate into AT1 type cells, and proliferate to promote re‐epithelialization.^142^ Following alveolar epithelial cell injury, infiltration of fibroblasts and inflammatory cells leads to release and activation of pro‐fibrotic mediators such as TGFβ and PDGF, resulting in matrix synthesis and accumulation.^143^ In addition, the alveolar epithelium regulates production of urokinase and plasminogen activator inhibitor 1 (PAI1) and thereby controls the coagulation and fibrinolysis on the alveolar surface.^144^ Both hemorrhage and fibrin deposition in the alveolar space and microvasculature is reported to be associated SARS‐CoV‐2 pathology implying the role of alveolar epithelium in promoting the coagulation disorders in COVID‐19.

TGF‐β has been proposed as a potential therapeutic target in the treatment of COVID‐19 ^145^ and previous studies suggest that epithelial TGF‐β1 acts as a principal trigger regulating lung injury and fibrosis.^53^ Over expressing TGF‐β1 in vivo results in progressive pulmonary fibrosis ^146^ and TGF‐β increases expression of the TGF‐β activating integrin αvβ6.^74^ This upregulation of TGFβ through αvβ6 may suppress alveolar macrophage mediated type I interferon responses and thereby increase the chance of a persistent viral infection.^147^ The SARS‐CoV‐2 spike protein contains an RGD integrin‐binding domain close to the ACE2 binding region, which could potentially facilitate binding to RGD‐binding integrins,^148^ which includes several TGF‐β ‐activating integrins. Our recent data demonstrated that SARS‐CoV‐2 is able to bind αvβ3 and αvβ6 integrins to facilitate internalization into lung epithelial cells, which may be associated severe pathology associated COVID 19.^20^ These findings suggest intriguing roles of lung epithelial cells involvement in COVID‐19‐induced ARDS and pulmonary fibrosis.

## 9 POTENTIAL FIBROBLAST MEDIATED MECHANISMS IN COVID‐19 FACILITATED PULMONARY FIBROSIS

As discussed previously, fibroblasts play a major role in tissue repair, following tissue injury fibroblasts proliferate and differentiate into myofibroblasts and they modulate extracellular matrix (ECM) volume. Myofibroblasts produce dense ECM compared to fibroblasts and having α‐smooth muscle actin causes spatial reorganization of collagen fibrils, leading to stiffer ECM.^50^ As discussed above, TGF‐β can regulate fibroblast proliferation,^87^, ^88^ transdifferentiation to a contractile myofibroblast phenotype,^89^, ^90^, ^91^, ^92^ and can cause the production and deposition of ECM proteins.^87^, ^93^

Covid‐19 induced fibrotic changes in the lung may alter the biomechanics of the lung resulting in lung tissue stiffening, similar to pulmonary fibrosis. Additionally, it has been suggested that SARS‐CoV‐2 takes advantage of the altered mechanical properties evident in the aged lung that results from fibroblast dysfunction.^149^ Cytoskeletal rearrangement plays a major role in promoting cell‐cell spread of the virus.^150^ Furthermore, integrins, which are well known as a connecting link between the cytoskeleton and the ECM, can activate fibroblasts, macrophage phagocytosis, modulate endothelial barrier function and can directly activate latent TGFβ induced pro‐fibrotic pathways.^151^ Ultimately, stiffening of lung tissue will hinder gas exchange and eventually result in declining lung function, dyspnea, and exercise intolerance.

Finally, severe inflammation and the “cytokine storm”, has been proposed to be involved in COVID‐19 pathogenesis,^152^ although this continues to be a subject of controversy ^153^ and the relative importance of inflammatory cytokines in COVID‐19 is still unclear.^154^ While recent work has shown that cytokine levels in severe case of COVID‐19 are lower than those associated with ARDS unrelated to COVID‐19, sepsis and chimeric antigen receptor (CAR) T‐cell induced cytokine release syndrome, elevated levels of a number of inflammatory markers, particularly IL6, in severe cases of COVID‐19 are found to predict the need for mechanical ventilation.^155^, ^156^, ^157^ A number of randomized, controlled trials have reported the use of monoclonal antibodies that inhibit both membrane‐bound and soluble IL6 receptors in COVID‐19 patients with mixed results as they also included less severely ill patients and excluded patients receiving respiratory support.^158^, ^159^, ^160^, ^161^, ^162^, ^163^ The most recent of these, REMAP‐CAP, reported improved outcomes, including survival, in critically ill adult COVID‐19 patients who were receiving organ support in ICUs at the time of treatment with the interleukin‐6 receptor antagonists tocilizumab and sarilumab.^164^ As discussed earlier in this article, inflammatory cytokines including IL6 can have a profound effect on the pro‐fibrotic actions of fibroblasts.^101^, ^102^, ^103^, ^105^, ^106^, ^107^ As a result, IL6 may play a crucial role in the development of fibrotic changes in the lungs of COVID‐19 patients, in addition to its potential role in the acute phase response to infection.

The clearest evidence of a role of severe inflammation in COVID‐19 pathogenesis derives from the RECOVERY trial which demonstrated that treatment of hospitalized COVID‐19 patients with the potent anti‐inflammatory corticosteroid, dexamethasone, results in a significant reduction in 28‐day mortality ^165^ data subsequently supported by three other trials.^166^, ^167^, ^168^ Interestingly, dexamethasone treatment is also found to be beneficial only in patients receiving invasive mechanical ventilation or receiving oxygen without invasive mechanical ventilation with no evidence of benefit among patients who were not receiving respiratory support. It remains to be seen whether therapeutic strategies designed to limit inflammation generally, or IL6 specifically, might be beneficial in limiting the fibrogenic response in severe COVID‐19, and whether early intervention will prevent the development of persistent interstitial fibrosis which characterized previous SARS and MERS pandemics.

**FIGURE 1:**
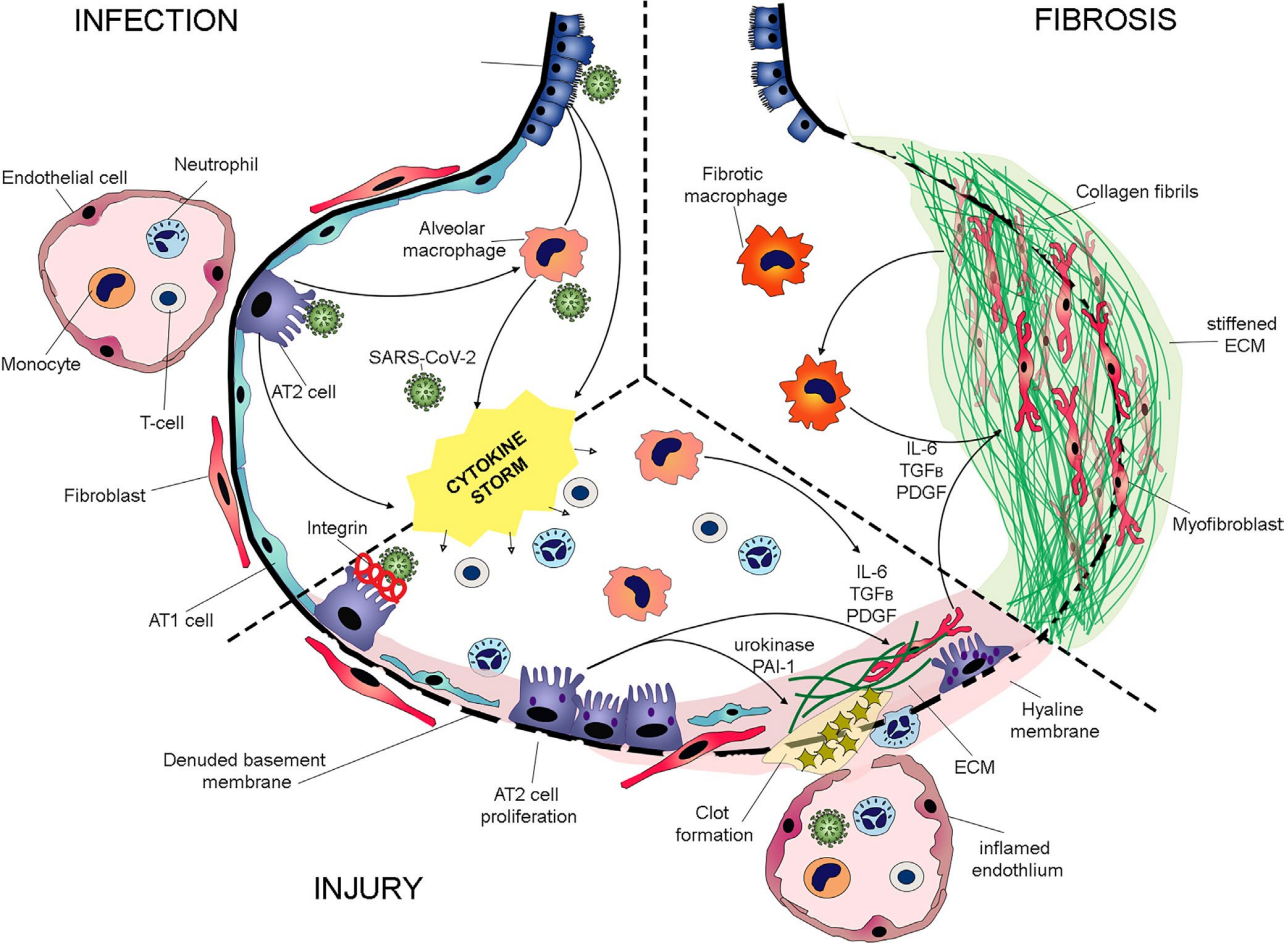
Proposed mechanism of SARS‐CoV‐2‐associated fibrosis in the lung. INFECTION with SARS‐CoV‐2 causes damage to the alveolar epithelium and induces production of epithelial and macrophage derived inflammatory and immune cytokines leading to lung INJURY. Activated inflammatory cells and damaged epithelial cells contribute to the denudation of the basement membrane leading to migration and proliferation of interstitial fibroblasts in the alveolar space in response to TGFb, PDGF and IL‐6. SARS‐CoV‐2 infection also injures endothelial cells resulting in hemorrhage and leakage of plasma into the alveolus. In response to urokinase and PAI‐1 release from the damaged alveolar epithelium, coagulation pathways are activated leading to fibrin deposition. Persistent alveolar activation of TGFb, release of PDGF and IL‐6 from alveolar epithelia cells, immune cells and myofibroblasts leads to proliferation of myofibroblasts and development of FIBROSIS

There is now clear emerging evidence that COVID‐19 can lead to fibrotic changes in the lungs and in this review we have tried to bring together the existing knowledge of potential mechanisms that might link initial infection to the development of lung tissue remodeling. We have summarized this knowledge and tried to illustrate the potential interplay between pathways discussed above in Figure 1. There are numerous potential ways in which SARS‐CoV‐2 might promote fibrogenesis including activation of inflammatory pathways, injury to the alveolar epithelium and vascular changes. More research is desperately needed to fully delineate the underlying pathogenic mechanisms that drive COVID‐19‐induced lung fibrosis.

## 10 PULMONARY FIBROSIS IN COVID‐19: STABLE OR PROGRESSIVE?

One year in to the COVID‐19 pandemic, there is mounting evidence to suggest that many COVID‐19 patients develop fibrotic sequelae and alterations in their lung function indicative of restrictive lung disease.^29^, ^34^, ^121^, ^123^, ^124^, ^125^, ^126^, ^127^, ^128^ It is still too early to know whether such changes occur purely as a transient response to viral infection and will spontaneously resolve with time, however, data collected thus far suggests that fibrosis persists for many months after the infection has resolved.^34^, ^136^, ^137^ A crucial question in the management and treatment of such patients in the years to come is whether post‐COVID‐19 fibrotic changes in the lung are stable once they have developed or are progressive, as in fibrotic lung diseases such as IPF.

There are many factors that might impact whether post‐COVID‐19 lung fibrosis has the potential to become progressive and life‐limiting. Genetics is likely to play a fundamental role. Genetic studies have highlighted genes involved with innate antiviral defenses, inflammatory lung injury and the ABO blood‐group system are associated with life‐threatening COVID‐19.^169^, ^170^ While no studies to date have studied genetic associations with post‐COVID‐19 fibrosis specifically, genome‐wide association studies have highlighted numerous genes associated with the development of pulmonary fibrosis.^42^, ^43^, ^44^ This raises the possibility that COVID‐19 infection in individuals with genetic alterations known to be associated with the development of lung fibrosis may result in a more progressive post‐COVID‐19 fibrosis. Prospective genome‐wide studies of individuals that develop fibrotic lung sequelae following COVID‐19 infection will shed light on the role that genetics plays in driving progressive or stable post‐COVID‐19 fibrosis.

Increased age is a key risk factor for both pulmonary fibrosis and COVID‐19,^6^, ^171^, ^172^, ^173^, ^174^ and could therefore be a contributing factor in whether post‐COVID‐19 fibrosis becomes progressive. Increased age is associated with stiffening of the lung parenchyma,^175^, ^176^ which could have important implications for TGFβ activation and the development of lung fibrosis.^53^ Age also affects the pro‐fibrotic potential of lung fibroblasts. Fibroblasts isolated from aged mice have reduced Thy‐1 expression, which is associated with a pro‐fibrotic phenotype,^177^, ^178^ plus reduced apoptosis and increased responses to TGFβ.^179^ Furthermore, culturing fibroblasts and lung epithelial cells on decellularized aged ECM leads to alterations in the composition of ECM deposited by the cells.^180^ Crucially, viral‐induced lung injury results in exacerbated lung fibrosis in aged mice.^181^, ^182^, ^183^ The role that increased age plays in the development and progression of COVID‐19‐associated fibrotic changes requires further study.

Obesity and metabolic syndrome are common risk factors for COVID‐19.^7^, ^184^, ^185^ Both type 1 and type 2 diabetes are associated with significantly increased risk of mortality from COVID‐19.^184^, ^186^ Similarly, patients with pulmonary fibrosis are often overweight ^187^, ^188^ and are more like to present with a clinical history of hypertension or diabetes, suggestive of metabolic syndrome.^189^ Furthermore, increased body mass index (BMI) is associated with a increased risk of developing ARDS in at‐risk patients.^190^ While direct evidence showing that obesity and/or alterations in metabolism contributes to fibrogenesis in COVID‐19 is lacking, there are several studies suggesting a mechanistic link with pulmonary fibrosis.^181^, ^191^, ^192^, ^193^ At a cellular level interrupting the signaling of peroxisome proliferator activated receptor gamma co‐activator 1‐alpha (PGC1α), a transcriptional co‐activator with important roles in regulating metabolism, enhances the contractility of fibroblasts and causes them to deposit greater amounts of collagen I and fibronectin.^194^ Similarly, reduced expression of PTEN, a protein that controls the metabolism of glucose and fatty acids, causes fibroblast‐myofibroblasts transdifferentiation and collagen production.^195^ Moreover, the anti‐diabetic drug Metformin can inhibit TGFβ‐induced fibrotic responses in lung fibroblasts in vitro and accelerate the resolution of experimental pulmonary fibrosis.^196^ Importantly, Metformin is associated with reduced mortality in COVID‐19 patients, particularly in women.^197^, ^198^ This supports the hypothesis that metabolic alterations are involved in COVID‐19 pathogenesis, however, the relative role of such alterations in driving either stable or progressive fibrosis requires further research.

## 11 CONCLUDING REMARKS

While viral infection can cause viral‐induced fibrosis ^116^ a clear association between viral infection and progressive fibrosis is still unclear. There is mounting evidence that fibrotic changes and interstitial lung abnormalities may result from COVID‐19 infection in some cases, however, how these changes develop and whether the fibrosis is stable or progressive is unknown. More research is urgently needed to (a) confirm that COVID‐19 can result in fibrotic lung disease, (b) establish the prevalence and epidemiology of such changes, and (c) delineate the cellular and molecular mechanisms driving fibrotic changes following SARS‐CoV‐2 infection. Through drawing on the vast existing literature from the field of pulmonary fibrosis we hypothesize that such fibrotic changes will involve both epithelial and fibroblast‐mediated mechanisms. Furthermore, our knowledge of the mechanisms underpinning lung fibrosis suggests that genetics, age and metabolic alterations may all play a role in driving the fibrotic phenotype and ultimately the long‐term outcome for post‐COVID‐19 lung fibrosis.

## CONFLICT OF INTEREST

Prof Jenkins has received sponsored research agreements from: Biogen, Galecto, GSK, Medimmune. Plus personal fees from: Galapagos, Heptares, Boehringer Ingelheim, Pliant, Roche/Intermune, Medimmune, Pharmakea, Bristol Myers Squibb, Chiesi, Roche/Promedior. Plus collaborative awards from: RedX and Nordic Biosciences. Finally, he is an advisory board member for NuMedii.
